# Supplementary material for: Relationship between self-reported listening and communication difficulties and executive function: a protocol for a systematic review and meta-analysis
Source: BMJ Open. 2023 Nov 8;13(11):e071225. doi: 10.1136/bmjopen-2022-071225 (PMC10632861; doi:10.1136/bmjopen-2022-071225)
Supplement: Supplementary data [file bmjopen-2022-071225supp001.pdf]

**Search strategies**
**MEDLINE & PsychINFO (OVID)** exp = explode the search term to include narrower more specific terms, .af. = search all fields in the document
1. exp Hearing Loss/<br>2. exp Hearing/<br>3. exp Self Report/<br>4. (self report* or self-report* or questionnaire).af.<br>5. exp Cognition/<br>6. cogniti* or executive or attention* or memory).af.<br>7. (inhibit* or updat* or shift*) .af.<br>8. 1 or 2<br>9. 3 or 4<br>10. 5 or 6 or 7<br>11. 8 and 9 and 10
**EMBASE (OVID)**
1. exp Hearing Disorders/<br>2. hearing.mp.<br>3. exp Self-Report/<br>4. exp Cognition/<br>5. 1 or 2<br>6. 3 and 4 and 5
**PubMed & Scopus**
1. hearing loss[MeSH Major Topic]<br>2. hearing[MeSH Major Topic]<br>3. self report[MeSH Major Topic]<br>4. self report*[Title/Abstract]<br>5. questionnaire[Title/Abstract]<br>6. cognition[MeSH Major Topic]<br>7. cogniti*[Title/Abstract]<br>8. executive[Title/Abstract]<br>9. attention*[Title/Abstract]<br>10. memory[Title/Abstract]<br>11. inhibi*[Title/Abstract]<br>12. updat*[Title/Abstract]<br>13. switch*[Title/Abstract]<br>14. 1 or 2<br>15. 3 or 4 or 5<br>16. 6 or 7 or 8 or 9 or 10 or 11 or 12 or 13<br>17. 14 and 15 and 16
**ASSIA (via ProQuest)**
1. su(Hearing Loss)<br>2. su(Hearing)<br>3. su(self-Report)<br>4. noft(self-report*)<br>5. noft(questionnaire)<br>6. su(cognition)<br>7. noft(cogniti*)<br>8. noft(executive)<br>9. noft(attention*)<br>10. noft(memory)<br>11. noft(inhibit*)

12. noft(update*)<br>13. noft(shift*)<br>14. 1 or 2<br>15. 3 or 4 or 5<br>16. 6 or 7 or 8 or 9 or 10 or 11 or 12 or 13<br>14. 14 and 15 and 16
**Web of Science**
1. (Hearing loss)<br>2. (Hearing)<br>3. (Self report)<br>4. KP=(Self report*)<br>5. KP=(questionnaire)<br>6. KP=(cogniti*))<br>7. KP=(attention*))<br>8. KP=(memory)<br>9. TS=(cognition)<br>10. 1 or 2<br>11. 3 or 4 or 5<br>12. 6 or 7 or 8 or 9<br>13. 10 and 11 and 12
**CINAHL (via EBSCO)**
1. MH hearing loss<br>2. deafness<br>3. hearing impairment<br>4. deaf<br>5. hard of hearing<br>6. MH self-report measures<br>7. self-report questionnaire<br>8. MH cognition<br>9. cognitive function<br>10. TX cogniti*<br>11. TX executive<br>12. TX attention*<br>13. TX memory<br>14. 1 or 2 or 3 or 4 or 5<br>15. 6 or 7<br>16. 8 or 9 or 10 or 11 or 12 or 13<br>17. 14 and 15 and 16
