# Supplementary material for: Relationship between self-reported listening and communication difficulties and executive function: a protocol for a systematic review and meta-analysis
Source: BMJ Open. 2023 Nov 8;13(11):e071225. doi: 10.1136/bmjopen-2022-071225 (PMC10632861; doi:10.1136/bmjopen-2022-071225)
Supplement: Author's manuscript [file bmjopen-2022-071225.draft_revisions.pdf]

BMJ Open is committed to open peer review. As part of this commitment we make the peer review history of every article we publish publicly available.

When an article is published we post the peer reviewers' comments and the authors' responses online. We also post the versions of the paper that were used during peer review. These are the versions that the peer review comments apply to.

The versions of the paper that follow are the versions that were submitted during the peer review process. They are not the versions of record or the final published versions. They should not be cited or distributed as the published version of this manuscript.

BMJ Open is an open access journal and the full, final, typeset and author-corrected version of record of the manuscript is available on our site with no access controls, subscription charges or pay-per-view fees (http://bmjopen.bmj.com).

If you have any questions on BMJ Open's open peer review process please email info.bmjopen@bmj.com

BMJ Open

The relationship between self-reported listening and communication difficulties and executive function: A protocol for a systematic review and meta-analysis

Journal:	*BMJ Open*
Manuscript ID	bmjopen-2022-071225
Article Type:	Protocol
Date Submitted by the Author:	03-Jan-2023
Complete List of Authors:	Stacey, Jemaine; Nottingham Trent University, Psychology<br>Danielsson, Henrik; Linköping University, Department of Behavioural Sciences and Learning<br>Heinrich, Antje ; The University of Manchester<br>Batinović, Lucija; Linköping University, Department of Behavioural Sciences and Learning<br>Ingo, Elisabeth; Linköping University, Department of Behavioural Sciences and Learning<br>Henshaw, Helen; University of Nottingham School of Medicine, Hearing Sciences, Mental Health and Clinical Neurosciences; NIHR Nottingham Biomedical Research Centre, National Institute for Health & Care Research (NIHR)
Keywords:	Audiology < OTOLARYNGOLOGY, GERIATRIC MEDICINE, GENERAL MEDICINE (see Internal Medicine)

SCHOLARONE™
Manuscripts

The relationship between self-reported listening and communication difficulties and executive function: A protocol for a systematic review and meta-analysis

Jemaine E. Stacey[1] https://orcid.org/0000-0003-4035-712X Henrik Danielsson[2] https://orcid.org/0000-0002-0446-0827 Antje Heinrich[3] https://orcid.org/0000-0002-5940-622X Lucija Batinović[2], Emil Holmer[2] https://orcid.org/0000-0002-1896-8250, Elisabeth Ingo[2] https://orcid.org/0000-0003-4025-6345 and Helen Henshaw[4,5] https://orcid.org/0000-0002-0547-4403

[1]Nottingham Trent University, NTU Psychology, 50 Shakespeare St, Nottingham UK.
[2]Department of Behavioural Sciences and Learning, Linköping University, Linköping, Sweden.
[3]Manchester Centre for Audiology and Deafness (ManCAD), School of Health Sciences, University of Manchester, UK.
[4]National Institute for Health & Care Research (NIHR) Nottingham Biomedical Research Centre, Ropewalk House, 113 The Ropewalk, Nottingham, UK.
[5]Hearing Sciences, Mental Health and Clinical Neurosciences, School of Medicine, University of Nottingham, Nottingham, UK.

Keywords: hearing loss, listening and communication difficulties, self-report, cognition, executive function, memory, attention

Corresponding author: Dr Helen Henshaw, NIHR Nottingham Biomedical Research Centre, Ropewalk House, 113 The Ropewalk, Nottingham, NG1 5DU, UK.
Email: helen.henshaw@nottingham.ac.uk

Abstract

Introduction.
Listening and communication difficulties can limit people's participation in activity and adversely affect their quality of life. Hearing, as well as listening and communication difficulties can be measured either by using behavioural tests or self-report, and the outcomes are not always closely linked. The association between behaviourally measured and self-reported hearing is strong, whereas, the association between behavioural and self-reported measures of listening and communication difficulties is much weaker, suggesting they assess slightly different aspects of listening. While behavioural measures of listening and communication difficulties have been associated with poorer cognitive performance including executive functions, the same association has not always been shown for self-report measures. The objective of this systematic review and meta-analysis is to understand the relationship between executive function and self-reported listening and communication difficulties in adults with hearing loss, and where possible, potential covariates of age and pure-tone audiometric thresholds.

Methods and Analysis. Studies will be eligible for inclusion if they report data from both a self-report measure of listening difficulties and a behavioural measure of executive function. Eight databases are to be searched: MEDLINE (via Ovid SP), EMBASE (via Ovid SP), PsycINFO (via Ovid SP), ASSIA (via ProQuest), Cumulative Index to Nursing and Allied Health Literature or CINAHL (via EBSCO Host), Scopus, PubMed, and Web of Science (Science and Social Science Citation Index). The Weight of Evidence (WoE) framework will be used to assess risk of bias for included studies. Results will be synthesised primarily using

For peer review only - http://bmjopen.bmj.com/site/about/guidelines.xhtml

a meta-analysis, and where sufficient quantitative data are not available, a narrative synthesis will be carried out to describe key results. **Ethics and dissemination.** No ethical issues are foreseen. Data will be disseminated via academic publication and conference presentations. Findings may also be published in scientific newsletters and magazines. PROSPERO registration number CRD42022293546.

Article Summary

Strengths and limitations of this study

- This systematic review is the first to investigate the relationship between self-reported listening and communication difficulties and executive function using meta-analysis to synthesise the available evidence.
- It uses an established framework (ICF) and taxonomy (CHC-M) to define target domains and measures of listening and communication difficulties and executive functions respectively.
- Grey literature (including unpublished study results) will be included.
- This Protocol has been reported in accordance with the PRISMA-P statement
- Only studies available in English are eligible for inclusion

Introduction

Listening and communication are crucial for a healthy life and difficulties in communication can limit people's participation and adversely affect their quality of life. Hearing loss plays a fundamental role in determining a person's ability to listen and communicate, although research over the years has shown that there are other factors, beyond hearing loss, that are also important. Both hearing, and listening and communication, can be measured using behavioural measures such as pure-tone audiograms and speech-in-noise tests, or via self-report questionnaires. Behavioural and self-report measures of hearing are generally well correlated, and behavioural measures of hearing are often well correlated with both behavioural and self-report measures of listening and communication, i.e. questionnaires. When a listener has a behaviourally measured hearing loss, it is likely that they will also experience and report difficulties with listening and communicating.

Effective communication, which relies on good hearing, is instrumental for a high level of functioning and good quality of life. [1] Kiessling et al. proposed a cascade linking hearing to effective communication, which in turn can be mapped to the ICF Framework (core set for hearing loss). [2] Both frameworks are displayed in Table 1:

Table 1 Frameworks describing hearing and effective communication

**Kiessling et al. [2]**	**ICF Framework**	
	BODY FUNCTIONS<br>= physiological functions of body systems	
**Hearing:** a passive function that provides access to the auditory world via the perception of sound	b230	Hearing functions:
	ACTIVITIES AND PARTICIPATION	

For peer review only - http://bmjopen.bmj.com/site/about/guidelines.xhtml

	= execution of a task or action by an individual and involvement in a life situation	
**Listening:** the process of hearing with intention and attention	d115	Listening:
**Comprehending:** the reception of information, meaning or intent	d310	Communicating with – receiving – spoken messages
**Communication:** the bi-directional transfer of information, meaning or intent between two or more people	d350	Conversation:

On the other hand, considering the activities and participation domain of listening and communication, behavioural and self-report measures are less robustly correlated. This is highlighted by the fact that two individuals who experience the same pure-tone average audiometric thresholds can experience and report substantially different degrees of listening and communication difficulties [3]. One interpretation of this result could be that they assess slightly different concepts and or highlight different contributing factors. One of those contributing factors whose role still remains to be fully understood is cognition [4].

Cognition has a complex relationship with auditory function depending on whether it is considered on the function (hearing) or activities/participation (listening/communication) level. Specifically, hearing loss, both behaviourally measured and self-reported, has been shown to be associated with poorer cognitive performance across a range of cognitive domains including; global cognition, episodic memory, processing speed, semantic memory, visuospatial ability, executive functions, as well as cognitive impairment and dementia [5]. Indeed, Marrone and colleagues [6] reported that adults reporting any trouble hearing were at nearly four times higher odds of reporting increased confusion and memory loss and half as likely to report good general health compared to adults reporting no hearing difficulty. These results are important to acknowledge because hearing loss has been identified as the leading potentially modifiable risk factor for dementia in midlife [7].

For listening and communication, on the other hand, the type of assessment appears to play a role. For behavioural measures, the role of cognition for the ability to perceive speech (and in particular, speech in noise) has been reliably demonstrated for individuals with hearing loss, and this relationship is robust even when taking into consideration individuals' age and objective hearing levels (pure-tone average audiometric thresholds) [8]. Note that the cognitive ability most commonly assessed in studies of speech perception in noise is working memory. Other abilities such as attention and executive function are less regularly assessed and less robustly found to link to speech perception in noise. One reason for the less robust link might be that the speech in noise perception task needs to be a particular type or of more complexity in order to necessitate attentional and executive functions.

For self-report measures of listening or communication difficulties in quiet and in noise on the other hand the role of cognition is much less clear and a clear link with cognition is not always shown [8]. It is unclear why this link is so variable. Again, the cognitive ability most likely to be assessed is working memory. Maybe the listening situations most commonly assessed with self-report measures of communication are not of the type that require working memory or are more complex listening situations that would necessitate the involvement of executive functions. This idea would make sense given that listening and communicating in complex and noisy environments draws upon the ability to shut out distractions and maintain

For peer review only - http://bmjopen.bmj.com/site/about/guidelines.xhtml

focus. And thus it is conceivable that differences in *executive functions,* may play a key role in the variation of individual experiences of listening and communication difficulties, regardless of absolute hearing levels.

Executive functions refer to "higher order cognitive processes that control lower level cognitive processes in the service of goal-directed behaviour" (p.186). [9] They enable the ability to think before acting, plan, meet novel and unanticipated challenges, resist temptations and maintain focus. [10] According to Miyake and Friedman [11], there are three core executive functions: mental-set shifting (*shifting*), information updating and monitoring (*updating*), and inhibition of prepotent responses (*inhibition*). Indeed, there is emerging evidence from largescale Cohort studies that individuals with self-reported hearing loss exhibit significantly poorer performance on tests of flexibility, psychomotor speed and executive function. [12] Similarly, a systematic review of tinnitus research found individuals who reported tinnitus had poorer performance on measure of executive function compared with individuals who did not. [13] Subsequent empirical research showed that for a population of adults with tinnitus, those reporting that their tinnitus was bothersome showed poorer performance on measures of executive function compared with those reporting non-bothersome tinnitus. [14]

In this review, we aim to synthesise the evidence assessing the relationship between self-reported listening and communication difficulties and objective measures of executive function, whilst controlling (where possible) for the potentially confounding factors of age and pure-tone audiometric hearing thresholds. To our knowledge, this independent relationship has yet to be extensively examined, despite data pertaining to both executive functions and self-reported listening and communication difficulties often being reported as part of wider research study methods.

When considering measures of self-reported listening and communication difficulties, it is important to clearly define what we mean, as there are well over a hundred self-report measures pertaining to listening [15], and only a subset will be relevant to our current research question. For this reason, we adopt definitions of listening difficulties provided by the International Classification of Functioning and Disease (ICF) as *activity limitations* and *participation restrictions* arising from hearing loss, and narrow our focus to self-report measures that align with ICF core set for hearing loss domains of *listening* (d115), *communicating with – receiving – spoken messages* (d310) and *conversation* (d350). Similarly, to definitively identify executive function domains and classify behavioural measures of executive function as either shifting, updating or inhibition, we will use the Cattell-Horn-Carroll-Miyake (CHC-M) taxonomy. [16]

Review questions

Primary research question
Is there an association between self-reported listening and communication difficulties and performance on behavioural measures of executive function in adults with hearing loss?

Secondary research question
Is any association moderated by age and/or hearing loss (as measured using average pure-tone audiometric thresholds)?

For peer review only - http://bmjopen.bmj.com/site/about/guidelines.xhtml

Objectives

1. To review and synthesise evidence for the association between self-reported listening difficulties and performance on behavioural measures of executive function, in adults with hearing loss.

Methods and Analysis

Eligibility criteria

Participants

Adults with hearing loss (with or without hearing devices), aged 18 years and over with no reported cognitive decline. We will accept a qualitative definition of hearing loss as “mild’, ‘moderate’, ‘severe’ or ‘profound’, or a quantitative definition where the group average pure-tone audiometric threshold is classed as mild hearing loss or greater using the World Health Organisation (WHO) definition of mild (26-40 dB HL inclusive); moderate (41-60 dB HL inclusive); severe (61-80 dB HL inclusive), and profound (81+ dB HL). [17] Studies that report on mixed populations (e.g. children & adults or normal hearing participants and participants with hearing loss) will be included only if the data for the populations of interest are reported separately.

Intervention/interest

A correlation coefficient between self-reported listening or communication difficulties and executive function, either reported or calculated from other reported data.

Outcomes

Self-reported listening and communication difficulties can be measured by a single item or a questionnaire assessing the following International Classification of Functioning and Disease (ICF) core set for hearing loss domains of listening (d115), communicating with – receiving – spoken messages (d310) and conversation (d350).

At least one behavioural measure of executive function must be included, defined according to the CHC-M taxonomy as tasks that measure: updating (e.g. verbal N-back), shifting (e.g. Trail making part B), and inhibition (e.g. Stroop). [16]

Where available, demographic information about the population (age, hearing device, group description) and objectively measured hearing loss (average pure-tone audiometric thresholds) will also be examined as subgroup descriptors and/or potential moderator(s).

Study design

Cross-sectional, longitudinal, experimental, quasi-experimental, and observational studies will be included.

Information sources

Articles must be available in English. No restrictions on publication dates will be applied.

Databases to be searched (see Table 2 for search terms): MEDLINE (via Ovid SP), EMBASE (via Ovid SP), PsycINFO (via Ovid SP), ASSIA (via ProQuest), Cumulative Index to Nursing and Allied Health Literature or CINAHL (via EBSCO Host), Scopus, PubMed, and Web of Science (Science and Social Science Citation Index). Gray literature including PhD theses, unpublished datasets and conference proceedings are eligible for inclusion.

For peer review only - http://bmjopen.bmj.com/site/about/guidelines.xhtml

Unpublished data will be accessed by contacting the corresponding authors of identified records. Literature searches were carried out on 11.05.2022.

Article selection process

Two reviewers will independently screen titles and abstracts, and full texts of retrieved records, against the inclusion and exclusion criteria. If insufficient information is provided in the titles and abstracts to know if it should be included or if there is disagreement between the two reviewers, the article will be included in the full-text screening. Disagreement at the full-text screening will be resolved by a third reviewer.

Data extraction process

A data extraction form will be created and improved by pilot testing before data extraction starts. The data from each study will be extracted separately by two reviewers and then compared. A third reviewer will be involved if there is any disagreement. Article selection and data extraction will be carried out using Covidence review management software (https://www.covidence.org/).

Data items

The data to be extracted are: the aim, study design, setting, conflicts of interest, demographic information about the population (age, hearing device, group description), sample size, bibliographic information (publication year, authors, journal), correlation coefficients between: self-reported listening difficulties and executive function (and [if reported] between pure-tone audiometric thresholds and self-reported listening difficulties/executive function), type of executive function measure, type of self-reported listening difficulty measure, and (where relevant) procedure of pure-tone audiometric assessment, as well as documenting any missing outcome data. The authors will be contacted via email if sufficient detail is not reported. If data are only reported via figures, then WebPlotDigitizer (http:// arohatgi.info/ WebPlotDigitizer/ app/) will be used to extract data. A third reviewer will be involved if there is any disagreement between data extracted.

Study risk of bias assessment

Two reviewers will assess risk of bias for each study identified by using the Weight of Evidence (WoE) framework. If disagreements arise a third reviewer will be involved. The WoE framework includes assessment of methodological quality, methodological relevance, and topic relevance. For each category studies will be rated low, medium or high.

Data synthesis

Key study characteristics will be described, including; study design, sample size, type of executive function measures used. The effect to be synthesised is the relationship between self-reported listening difficulties and behavioural measures of executive function defined by correlation coefficients. If correlation coefficients cannot be calculated or extracted for meta-analysis, study authors will be contacted to request the required information. Subgroup analyses will examine (where reported) key factors of, age, category/measure of self-reported listening difficulty, type of executive function measure, and type of hearing device, and pure-tone audiometric thresholds. Meta-analyses will be conducted for subgroups where data for a minimum of n=5 effects are available.

The meta-analysis will be carried out using the correlation coefficient as the outcome measure. A random-effects model will be fitted to the data. We will calculate at maximum one correlation coefficient per type of executive function by type of listening difficulty. If a

For peer review only - http://bmjopen.bmj.com/site/about/guidelines.xhtml

study reports multiple different correlations, there is likely to be some level of dependency in the data that needs to be dealt with. To handle any dependency between effect sizes in the analyses, a multilevel random-effects meta-analysis approach, as recommended by Assink and Wibbelink, will be applied. [18] This approach includes one random effect for each study as an addition to the random effect for each effect size. Likelihood ratio tests will compare the fit of the multilevel model to the fit of the reduced models. If the multilevel random-effects analysis has better fit, it will be used in all analyses, otherwise the random effects model will be used.

The amount of heterogeneity (i.e., τ 2) will be estimated using the restricted maximum-likelihood estimator. [19] In addition to the estimate of τ 2, the Q-test for heterogeneity and the I^2 statistic will be reported. [20, 21] In case any amount of heterogeneity is detected (i.e., $\tau\hat{}2 > 0$, regardless of the results of the Q-test), a prediction interval for the true outcomes will also be provided [22]. Studentized residuals and Cook's distances will be used to examine whether studies can be defined as outliers and potentially influential in the context of the model. [23] Studies with a studentized residual larger than the $100 \times (1 - 0.05/(2 \times k))$th percentile of a standard normal distribution will be considered potential outliers (i.e., using a Bonferroni correction with two-sided $\alpha = 0.05$ for k studies included in the meta-analysis). Studies with a Cook's distance larger than the median plus six times the interquartile range of the Cook's distances will be considered influential. The analysis will be carried out using R and the metafor package. [24, 25]

The analysis for the secondary research question will be carried out in the same way as above, with the difference that meta-regressions will be used to investigate potential moderator effects. The moderators will be evaluated one by one, and if both get significant effects, they will be evaluated together. The meta regression procedure will follow the tutorial for meta regression on the metafor package home page. [26]

Reporting bias assessment

Funnel plots will be used to assess reporting bias. In addition, the funnel plot asymmetry will be evaluated with the rank correlation test and the regression test, using the standard error of the observed outcomes as predictor. [27, 28]

Ethics and Dissemination

This review does not raise any ethical issues. Results will be disseminated via scientific peer-reviewed journal articles, scientific magazines, and conference presentations.

Patient and Public Involvement

Patients or the public were not involved in the creation of this review protocol.

Study Design

MeSH terms will be used in relevant databases.

Table 2: Search terms for databases

**MEDLINE (OVID)** exp = explode the search term to include narrower more specific terms, .af. = search all fields in the document
1. exp Hearing Loss/
2. exp Hearing/
3. exp Self Report/
4. (self report* or self-report* or questionnaire).af.

For peer review only - http://bmjopen.bmj.com/site/about/guidelines.xhtml

5. exp Cognition/
6. (cogniti* or executive or attention* or memory).af.
7. (inhibit* or updat* or shift*) .af.
8. 1 or 2
9. 3 or 4
10. 5 or 6 or 7
11. 8 and 9 and 10

Author Contributions

HH, AH and HD developed the study. HH and AH created the search terms. JS, AH and HH wrote the review protocol and HD wrote the meta-analysis methods. AH, HD, EI, EH and LB provided critical feedback on drafts of the protocol.

Contact information

Jemaine E. Stacey
E-mail: Jemaine.stacey@ntu.ac.uk
Twitter: @DrJStacey

Henrik Danielsson
E-mail: henrik.danielsson@liu.se
Twitter: @henda52

Helen Henshaw
E-mail: helen.henshaw@nottingham.ac.uk
Twitter: @hlh1

Antje Heinrich
E-mail: antje.heinrich@manchester.ac.uk

Elisabeth Ingo
E-mail: elisabeth.ingo@liu.se

Emil Holmer
E-mail: emil.holmer@liu.se

Lucija Batinović
E-mail: lucija.batinovic@liu.se

Data statement

No datasets were generated or analysed for this protocol.

Funding

This study is funded by the National Institute for Health and Care Research (NIHR) [CDF-2018-11-ST2-016 and PB-PG-0816-20044], the NIHR Nottingham Biomedical Research Centre [BRC-1215-20003], the NIHR Manchester Biomedical Research Centre [NIHR BRC 20007] and by the Swedish Research Council grant 2017-06092. The views expressed are those of the author(s) and not necessarily those of the NIHR or the Department of Health and Social Care.

For peer review only - http://bmjopen.bmj.com/site/about/guidelines.xhtml

Competing interests
None declared

Acknowledgements: none.

Patient and Public involvement

Patients or the public were not involved in the creation of this protocol as it relates to secondary data.

Word count: 2491 words

References

1. Dalton DS, Cruickshanks KJ, Klein BEK, *et al.* The Impact of Hearing Loss on Quality of Life in Older Adults. *Gerontologist*, **43(5):** 661–668. doi:10.1093/geront/43.5.661
2. Kiessling J, Pichora-Fuller MK, Gatehouse S, et al. Candidature for and delivery of audiological services: Special needs of older people. *Int J Audiol* 2003;**42(2)**:S92-S101. doi:10.3109/14992020309074650
3. Manchaiah VK, Stephens D. Perspectives on defining 'hearing loss' and its consequences. *Hearing Balance Commun* 2013;**11(1)**:6-16. doi:10.3109/21695717.2012.756624
4. Heinrich A, Henshaw H, Ferguson MA. Only behavioral but not self-report measures of speech perception correlate with cognitive abilities. *Front Psychol* 2016;**7**:576. doi:10.3389/fpsyg.2016.00576
5. Loughrey DG, Kelly ME, Kelley GA, *et al*. Association of age-related hearing loss with cognitive function, cognitive impairment, and dementia: A systematic review and meta-analysis. *JAMA Otolaryngol Head Neck Surg* 2018;**144(2)**:115-126. doi:10.1001/jamaoto.2017.2513
6. Marrone N, Ingram M, Bischoff K, *et al*. Self-reported hearing difficulty and its association with general, cognitive, and psychosocial health in the state of Arizona, 2015. *BMC Public Health* 2019;**19(1)**:875. doi:10.1186/s12889-019-7175-5
7. Livingston G, Huntley J, Sommerlad A, *et al*. Dementia prevention, intervention, and care: 2020 report of the Lancet Commission. *The Lancet* 2020;**396(10248)**:413-446. doi:10.1016/S0140-6736(20)30367-6
8. Heinrich A, Henshaw H, Ferguson MA. The relationship of speech intelligibility with hearing sensitivity, cognition, and perceived hearing difficulties varies for different speech perception tests. *Front Psychol* 2015;**6**:782. doi:10.3389/fpsyg.2015.00782
9. Friedman NP, Miyake A. Unity and diversity of executive functions: Individual differences as a window on cognitive structure. *Cortex* 2017;**86**:186-204. doi:10.1016/j.cortex.2016.04.023
10. Diamond A. Executive functions. *Annu Rev Psychol* 2013;**64**:135. doi:10.1146/annurev-psych-113011-143750

For peer review only - http://bmjopen.bmj.com/site/about/guidelines.xhtml

11. Miyake A, Friedman NP, Emerson MJ, *et al*. The unity and diversity of executive functions and their contributions to complex "frontal lobe" tasks: A latent variable analysis. *Cogn Psychol* 2000;**41(1)**:49-100. doi:10.1006/cogp.1999.0734
12. Fields TN, Mueller KD, Koscik RL, *et al*. Self-reported hearing loss and longitudinal cognitive function in a cohort enriched with risk for alzheimer's disease. *J Alzheimer's Dis* 2020;**78(3)**:1109-17. doi:10.3233/JAD-200701
13. Clarke NA, Henshaw H, Akeroyd MA, *et al*. Associations between subjective tinnitus and cognitive performance: systematic review and meta-analyses. *Trends Hear* 2020;**24**:2331216520918416. doi:10.1177/2331216520918416
14. Clarke NA, Akeroyd MA, Henshaw H, *et al*. The executive disruption model of tinnitus distress. *Front Psycho* in press.
15. Akeroyd MA, Wright-Whyte K, Holman JA, *et al*. A comprehensive survey of hearing questionnaires: how many are there, what do they measure, and how have they been validated?. *Trials* 2015;**16(1)**:1-1. doi:10.1186/1745-6215-16-S1-P26
16. Webb SL, Loh V, Lampit A, *et al*. Meta-analysis of the effects of computerized cognitive training on executive functions: a cross-disciplinary taxonomy for classifying outcome cognitive factors. *Neuropsychol Rev* 2018;**28(2)**:232-50. doi:10.1007/s11065-018-9374-8
17. World health organisation. Deafness and hearing loss. 2022. https://www.who.int/news-room/fact-sheets/detail/deafness-and-hearing-loss Accessed: 6 July 2022.
18. Assink M, Wibbelink CJ. Fitting three-level meta-analytic models in R: A step-by-step tutorial. *Quant Method Psychol* 2016;**12(3)**:154-74.
19. Viechtbauer W. Bias and efficiency of meta-analytic variance estimators in the random-effects model. *J Edu Behav Stat* 2005;**30(3)**:261-93. doi:10.3102/10769986030003261
20. Higgins JP, Thompson SG. Quantifying heterogeneity in a meta-analysis. *Stat Med* 2002;**21(11):**1539-58.
21. Cochran WG. (1954). The combination of estimates from different experiments. *Biometrics* 1954;**10(1)**:101–129. doi:10.2307/3001666
22. Riley RD, Higgins JP, Deeks JJ. Interpretation of random effects meta-analyses. *BMJ* 2011;**342**. doi:10.1136/bmj.d549
23. Viechtbauer W, Cheung MW. Outlier and influence diagnostics for meta-analysis. *Res Synth Methods* 2010;**1(2):**112-25. doi:10.1002/jrsm.11
24. R Core Team. R: A language and environment for statistical computing. R Foundation for Statistical Computing. 2021. www.R-project.org/
25. Viechtbauer, W. Conducting meta-analyses in R with the Metafor package. *J Stat Softw* 2010;**36(3)**:1-48.
26. Viechtbauer W. The Metafor Package: A meta-analysis package for R. Metafor Project. 2021. www.metafor-project.org/doku.php. Accessed: 6 July 2022.
27. Begg CB, Mazumdar M. Operating characteristics of a rank correlation test for publication bias. *Biom* 1994;**1**:1088-1101. doi:10.2307/2533446
28. Sterne JA, Egger M. Regression methods to detect publication and other bias in meta-analysis. In: Rothstein HR, Sutton AJ, Borenstein M, eds. *Publication bias in meta-*

For peer review only - http://bmjopen.bmj.com/site/about/guidelines.xhtml

analysis: Prevention, assessment and adjustments. Wiley 2005. 99-110. doi:10.1002/0470870168

For peer review only

For peer review only - http://bmjopen.bmj.com/site/about/guidelines.xhtml

PRISMA-P (Preferred Reporting Items for Systematic review and Meta-Analysis Protocols) 2015 checklist: recommended items to address in a systematic review protocol*

Section and topic	Item No	Checklist item
**ADMINISTRATIVE INFORMATION**		
Title:		
Identification	1a	Identify the report as a protocol of a systematic review see title
Update	1b	If the protocol is for an update of a previous systematic review, identify as such n/a
Registration	2	If registered, provide the name of the registry (such as PROSPERO) and registration number see final line of abstract
Authors:		
Contact	3a	Provide name, institutional affiliation, e-mail address of all protocol authors; provide physical mailing address of corresponding author see both author affiliations and 'contact information' provided on page 8
Contributions	3b	Describe contributions of protocol authors and identify the guarantor of the review see 'author contributions' page 8 lines 4-7
Amendments	4	If the protocol represents an amendment of a previously completed or published protocol, identify as such and list changes; otherwise, state plan for documenting important protocol amendments N/A
Support:		
Sources	5a	Indicate sources of financial or other support for the review see 'funding' page 8 lines 37-43
Sponsor	5b	Provide name for the review funder and/or sponsor see 'funding' page 8 lines 37-43
Role of sponsor or funder	5c	Describe roles of funder(s), sponsor(s), and/or institution(s), if any, in developing the protocol page 8 lines 37-43
**INTRODUCTION**		
Rationale	6	Describe the rationale for the review in the context of what is already known page 2 line 21 onwards
Objectives	7	Provide an explicit statement of the question(s) the review will address with reference to participants, interventions, comparators, and outcomes (PICO) see page 4 'review questions' line 41 onwards AND the PICO is on page 5 lines 9-35
**METHODS**		
Eligibility criteria	8	Specify the study characteristics (such as PICO, study design, setting, time frame) and report characteristics (such as years considered, language, publication status) to be used as criteria for eligibility for the review see page 5 'eligibility criteria'
Information sources	9	Describe all intended information sources (such as electronic databases, contact with study authors, trial registers or other grey literature sources) with planned dates of coverage see 'information sources' page 5-6 line 43 onwards
Search strategy	10	Present draft of search strategy to be used for at least one electronic database, including planned limits, such that it could be repeated see table 1 on page 8-9
Study records:		

For peer review only

Data management	11a	Describe the mechanism(s) that will be used to manage records and data throughout the review page 6 lines 14-15
Selection process	11b	State the process that will be used for selecting studies (such as two independent reviewers) through each phase of the review (that is, screening, eligibility and inclusion in meta-analysis) see page 6 see "article selection process'
Data collection process	11c	Describe planned method of extracting data from reports (such as piloting forms, done independently, in duplicate), any processes for obtaining and confirming data from investigators see page 6 see "data extraction process"
Data items	12	List and define all variables for which data will be sought (such as PICO items, funding sources), any pre-planned data assumptions and simplifications page 6 see "data items"
Outcomes and prioritization	13	List and define all outcomes for which data will be sought, including prioritization of main and additional outcomes, with rationale page 5 23-27
Risk of bias in individual studies	14	Describe anticipated methods for assessing risk of bias of individual studies, including whether this will be done at the outcome or study level, or both; state how this information will be used in data synthesis see page 6 lines 32-35
Data synthesis	15a	Describe criteria under which study data will be quantitatively synthesised see data synthesis page 6 lines 38- page 7 line 1 onward
	15b	If data are appropriate for quantitative synthesis, describe planned summary measures, methods of handling data and methods of combining data from studies, including any planned exploration of consistency (such as I^2, Kendall's τ) page 7 lines 10-27
	15c	Describe any proposed additional analyses (such as sensitivity or subgroup analyses, meta-regression) page 6 lines 45-46
	15d	If quantitative synthesis is not appropriate, describe the type of summary planned page 2 line 1
Meta-bias(es)	16	Specify any planned assessment of meta-bias(es) (such as publication bias across studies, selective reporting within studies) see page 7 lines 30-32
Confidence in cumulative evidence	17	Describe how the strength of the body of evidence will be assessed (such as GRADE) see page 6 lines 32-35

*** It is strongly recommended that this checklist be read in conjunction with the PRISMA-P Explanation and Elaboration (cite when available) for important clarification on the items. Amendments to a review protocol should be tracked and dated.** **The copyright for PRISMA-P (including checklist) is held by the PRISMA-P Group and is distributed under a Creative Commons Attribution Licence 4.0.**

From: Shamseer L, Moher D, Clarke M, Ghersi D, Liberati A, Petticrew M, Shekelle P, Stewart L, PRISMA-P Group. Preferred reporting items for systematic review and meta-analysis protocols (PRISMA-P) 2015: elaboration and explanation. BMJ. 2015 Jan 2;349(jan02 1):g7647.

For peer review only

The relationship between self-reported listening and communication difficulties and executive function: A protocol for a systematic review and meta-analysis

Journal:	*BMJ Open*
Manuscript ID	bmjopen-2022-071225.R1
Article Type:	Protocol
Date Submitted by the Author:	19-Jul-2023
Complete List of Authors:	Stacey, Jemaine; Nottingham Trent University, Psychology<br>Danielsson, Henrik; Linköping University, Department of Behavioural Sciences and Learning<br>Heinrich, Antje ; The University of Manchester<br>Batinović, Lucija; Linköping University, Department of Behavioural Sciences and Learning<br>Holmer, Emil ; Linköping University, Department of Behavioural Sciences and Learning<br>Ingo, Elisabeth; Linköping University, Department of Behavioural Sciences and Learning<br>Henshaw, Helen; University of Nottingham School of Medicine, Hearing Sciences, Mental Health and Clinical Neurosciences; NIHR Nottingham Biomedical Research Centre, National Institute for Health & Care Research (NIHR)
**Primary Subject Heading**:	Ear, nose and throat/otolaryngology
Secondary Subject Heading:	Geriatric medicine, Ear, nose and throat/otolaryngology, Communication
Keywords:	Audiology < OTOLARYNGOLOGY, GENERAL MEDICINE (see Internal Medicine), Aged

SCHOLARONE™
Manuscripts

The relationship between self-reported listening and communication difficulties and executive function: A protocol for a systematic review and meta-analysis

Jemaine E. Stacey[1] https://orcid.org/0000-0003-4035-712X Henrik Danielsson[2] https://orcid.org/0000-0002-0446-0827 Antje Heinrich[3] https://orcid.org/0000-0002-5940-622X Lucija Batinović[2], Emil Holmer[2] https://orcid.org/0000-0002-1896-8250, Elisabeth Ingo[2] https://orcid.org/0000-0003-4025-6345 and Helen Henshaw[4,5] https://orcid.org/0000-0002-0547-4403

[1]Nottingham Trent University, NTU Psychology, 50 Shakespeare St, Nottingham UK.
[2]Department of Behavioural Sciences and Learning, Linköping University, Linköping, Sweden.
[3]Manchester Centre for Audiology and Deafness (ManCAD), School of Health Sciences, University of Manchester, UK.
[4]National Institute for Health & Care Research (NIHR) Nottingham Biomedical Research Centre, Ropewalk House, 113 The Ropewalk, Nottingham, UK.
[5]Hearing Sciences, Mental Health and Clinical Neurosciences, School of Medicine, University of Nottingham, Nottingham, UK.

Keywords: hearing loss, listening and communication difficulties, self-report, cognition, executive function, memory, attention

Corresponding author: Dr Helen Henshaw, NIHR Nottingham Biomedical Research Centre, Ropewalk House, 113 The Ropewalk, Nottingham, NG1 5DU, UK.
Email: helen.henshaw@nottingham.ac.uk

Abstract

Introduction.
Listening and communication difficulties can limit people's participation in activity and adversely affect their quality of life. Hearing, as well as listening and communication difficulties can be measured either by using behavioural tests or self-report, and the outcomes are not always closely linked. The association between behaviourally measured and self-reported hearing is strong, whereas, the association between behavioural and self-reported measures of listening and communication difficulties is much weaker, suggesting they assess slightly different aspects of listening. While behavioural measures of listening and communication difficulties have been associated with poorer cognitive performance including executive functions, the same association has not always been shown for self-report measures. The objective of this systematic review and meta-analysis is to understand the relationship between executive function and self-reported listening and communication difficulties in adults with hearing loss, and where possible, potential covariates of age and pure-tone audiometric thresholds.

Methods and Analysis. Studies will be eligible for inclusion if they report data from both a self-report measure of listening difficulties and a behavioural measure of executive function. Eight databases are to be searched: MEDLINE (via Ovid SP), EMBASE (via Ovid SP), PsycINFO (via Ovid SP), ASSIA (via ProQuest), Cumulative Index to Nursing and Allied Health Literature or CINAHL (via EBSCO Host), Scopus, PubMed, and Web of Science (Science and Social Science Citation Index). The JBI critical appraisal tool will be used to assess risk of bias for included studies. Results will be synthesised primarily using a meta-

For peer review only - http://bmjopen.bmj.com/site/about/guidelines.xhtml

analysis, and where sufficient quantitative data are not available, a narrative synthesis will be carried out to describe key results. **Ethics and dissemination.** No ethical issues are foreseen. Data will be disseminated via academic publication and conference presentations. Findings may also be published in scientific newsletters and magazines. PROSPERO registration number CRD42022293546.

Article Summary

Strengths and limitations of this study

- This systematic review is the first to investigate the relationship between self-reported listening and communication difficulties and executive function using meta-analysis to synthesise the available evidence.
- It uses an established framework (ICF) and taxonomy (CHC-M) to define target domains and measures of listening and communication difficulties and executive functions respectively.
- Grey literature (including unpublished study results) will be included.
- This Protocol has been reported in accordance with the PRISMA-P statement
- Only studies available in English are eligible for inclusion

Introduction

Listening and communication are crucial for a healthy life and difficulties in communication can limit people's participation and adversely affect their quality of life. Hearing loss plays a fundamental role in determining a person's ability to listen and communicate, although research over the years has shown that there are other factors, beyond hearing loss, that are also important. Both hearing, and listening and communication, can be measured using behavioural measures such as pure-tone audiograms and speech-in-noise tests, or via self-report questionnaires. Behavioural and self-report measures of hearing are generally well correlated, and behavioural measures of hearing are often well correlated with both behavioural and self-report measures of listening and communication, i.e. questionnaires. When a listener has a behaviourally measured hearing loss, it is likely that they will also experience and report difficulties with listening and communicating.

Effective communication, which relies on good hearing, is instrumental for a high level of functioning and good quality of life. [1] Kiessling et al. proposed a cascade linking hearing to effective communication, which in turn can be mapped to the ICF Framework (core set for hearing loss). [2] Both frameworks are displayed in Table 1:

Table 1 Frameworks describing hearing and effective communication

**Kiessling et al. [2]**	**ICF Framework**	
	BODY FUNCTIONS<br>= physiological functions of body systems	
**Hearing:** a passive function that provides access to the auditory world via the perception of sound	b230	Hearing functions:
	ACTIVITIES AND PARTICIPATION	

For peer review only - http://bmjopen.bmj.com/site/about/guidelines.xhtml

	= execution of a task or action by an individual and involvement in a life situation	
**Listening:** the process of hearing with intention and attention	d115	Listening:
**Comprehending:** the reception of information, meaning or intent	d310	Communicating with – receiving – spoken messages
**Communication:** the bi-directional transfer of information, meaning or intent between two or more people	d350	Conversation:

On the other hand, considering the activities and participation domain of listening and communication, behavioural and self-report measures are less robustly correlated. This is highlighted by the fact that two individuals who experience the same pure-tone average audiometric thresholds can experience and report substantially different degrees of listening and communication difficulties [3]. One interpretation of this result could be that they assess slightly different concepts and or highlight different contributing factors. One of those contributing factors whose role still remains to be fully understood is cognition [4].

Cognition has a complex relationship with auditory function depending on whether it is considered on the function (hearing) or activities/participation (listening/communication) level. Specifically, hearing loss, both behaviourally measured and self-reported, has been shown to be associated with poorer cognitive performance across a range of cognitive domains including; global cognition, episodic memory, processing speed, semantic memory, visuospatial ability, executive functions, as well as cognitive impairment and dementia [5]. Indeed, Marrone and colleagues [6] reported that adults reporting any trouble hearing were at nearly four times higher odds of reporting increased confusion and memory loss and half as likely to report good general health compared to adults reporting no hearing difficulty. These results are important to acknowledge because hearing loss has been identified as the leading potentially modifiable risk factor for dementia in midlife [7].

For listening and communication, on the other hand, the type of assessment appears to play a role. For behavioural measures, the role of cognition for the ability to perceive speech (and in particular, speech in noise) has been reliably demonstrated for individuals with hearing loss, and this relationship is robust even when taking into consideration individuals' age and objective hearing levels (pure-tone average audiometric thresholds) [8]. Note that the cognitive ability most commonly assessed in studies of speech perception in noise is working memory. Other abilities such as attention and executive function are less regularly assessed and less robustly found to link to speech perception in noise. One reason for the less robust link might be that the speech in noise perception task needs to be a particular type or of more complexity in order to necessitate attentional and executive functions.

For self-report measures of listening or communication difficulties in quiet and in noise on the other hand the role of cognition is much less clear and a clear link with cognition is not always shown [8]. It is unclear why this link is so variable. Again, the cognitive ability most likely to be assessed is working memory. Maybe the listening situations most commonly assessed with self-report measures of communication are not of the type that require working memory or are more complex listening situations that would necessitate the involvement of executive functions. This idea would make sense given that listening and communicating in complex and noisy environments draws upon the ability to shut out distractions and maintain

For peer review only - http://bmjopen.bmj.com/site/about/guidelines.xhtml

focus. And thus it is conceivable that differences in *executive functions,* may play a key role in the variation of individual experiences of listening and communication difficulties, regardless of absolute hearing levels.

Executive functions refer to "higher order cognitive processes that control lower level cognitive processes in the service of goal-directed behaviour" (p.186). [9] They enable the ability to think before acting, plan, meet novel and unanticipated challenges, resist temptations and maintain focus. [10] According to Miyake and Friedman [11], there are three core executive functions: mental-set shifting (*shifting*), information updating and monitoring (*updating*), and inhibition of prepotent responses (*inhibition*). Indeed, there is emerging evidence from largescale Cohort studies that individuals with self-reported hearing loss exhibit significantly poorer performance on tests of flexibility, psychomotor speed and executive function. [12] Similarly, a systematic review of tinnitus research found individuals who reported tinnitus had poorer performance on measure of executive function compared with individuals who did not. [13] Subsequent empirical research showed that for a population of adults with tinnitus, those reporting that their tinnitus was bothersome showed poorer performance on measures of executive function compared with those reporting non-bothersome tinnitus. [14]

In this review, we aim to synthesise the evidence assessing the relationship between self-reported listening and communication difficulties and objective measures of executive function, whilst controlling (where possible) for the potentially confounding factors of age and pure-tone audiometric hearing thresholds. To our knowledge, this independent relationship has yet to be extensively examined, despite data pertaining to both executive functions and self-reported listening and communication difficulties often being reported as part of wider research study methods.

When considering measures of self-reported listening and communication difficulties, it is important to clearly define what we mean, as there are well over a hundred self-report measures pertaining to listening [15], and only a subset will be relevant to our current research question. For this reason, we adopt definitions of listening difficulties provided by the International Classification of Functioning and Disease (ICF) as *activity limitations* and *participation restrictions* arising from hearing loss, and narrow our focus to self-report measures that align with ICF core set for hearing loss domains of *listening* (d115), *communicating with – receiving – spoken messages* (d310) and *conversation* (d350). Similarly, to definitively identify executive function domains and classify behavioural measures of executive function as either shifting, updating or inhibition, we will use the Cattell-Horn-Carroll-Miyake (CHC-M) taxonomy. [16]

Review questions

Primary research question
Is there an association between self-reported listening and communication difficulties and performance on behavioural measures of executive function in adults with hearing loss?

Secondary research question
Is any association moderated by age and/or hearing loss (as measured using average pure-tone audiometric thresholds)?

For peer review only - http://bmjopen.bmj.com/site/about/guidelines.xhtml

Objectives

1. To review and synthesise evidence for the association between self-reported listening difficulties and performance on behavioural measures of executive function, in adults with hearing loss.

Methods and Analysis

Eligibility criteria

Participants

Adults with hearing loss (with or without hearing devices), aged 18 years and over with no reported cognitive decline. We will accept a qualitative definition of hearing loss as "mild', 'moderate', 'severe' or 'profound', or a quantitative definition where the group average pure-tone audiometric threshold is classed as mild hearing loss or greater using the World Health Organisation (WHO) definition of mild (26-40 dB HL inclusive); moderate (41-60 dB HL inclusive); severe (61-80 dB HL inclusive), and profound (81+ dB HL). [17] Studies that report on mixed populations (e.g. children & adults or normal hearing participants and participants with hearing loss) will be included only if the data for the populations of interest are reported separately.

Intervention/interest

A correlation coefficient between self-reported listening or communication difficulties and executive function, either reported or calculated from other reported data.

Outcomes

Self-reported listening and communication difficulties can be measured by a single item or a questionnaire assessing the following International Classification of Functioning and Disease (ICF) core set for hearing loss domains of listening (d115), communicating with – receiving – spoken messages (d310) and conversation (d350).

At least one behavioural measure of executive function must be included, defined according to the CHC-M taxonomy as tasks that measure: updating (e.g. verbal N-back), shifting (e.g. Trail making part B), and inhibition (e.g. Stroop). [16]

Where available, demographic information about the population (age, hearing device, group description) and objectively measured hearing loss (average pure-tone audiometric thresholds) will also be examined as subgroup descriptors and/or potential moderator(s).

Study design

Cross-sectional, longitudinal, experimental, quasi-experimental, and observational studies will be included.

Information sources

Articles must be available in English. No restrictions on publication dates will be applied.

Databases to be searched (see Table 2 for search terms): MEDLINE (via Ovid SP), EMBASE (via Ovid SP), PsycINFO (via Ovid SP), ASSIA (via ProQuest), Cumulative Index to Nursing and Allied Health Literature or CINAHL (via EBSCO Host), Scopus, PubMed, and Web of Science (Science and Social Science Citation Index). Gray literature including PhD theses, unpublished datasets and conference proceedings are eligible for inclusion.

For peer review only - http://bmjopen.bmj.com/site/about/guidelines.xhtml

Unpublished data will be accessed by contacting the corresponding authors of identified records. Literature searches were carried out on 11.05.2022.

Article selection process

Two reviewers will independently screen titles and abstracts, and full texts of retrieved records, against the inclusion and exclusion criteria. If insufficient information is provided in the titles and abstracts to know if it should be included or if there is disagreement between the two reviewers, the article will be included in the full-text screening. Disagreement at the full-text screening will be resolved by a third reviewer.

Data extraction process

A data extraction form will be created and improved by pilot testing before data extraction starts. The data from each study will be extracted separately by two reviewers and then compared. A third reviewer will be involved if there is any disagreement. Article selection and data extraction will be carried out using Covidence review management software (https://www.covidence.org/).

Data items

The data to be extracted are: the aim, study design, setting, conflicts of interest, demographic information about the population (age, hearing device, group description), sample size, bibliographic information (publication year, authors, journal), correlation coefficients between: self-reported listening difficulties and executive function (and [if reported] between pure-tone audiometric thresholds and self-reported listening difficulties/executive function), type of executive function measure, type of self-reported listening difficulty measure, and (where relevant) procedure of pure-tone audiometric assessment, as well as documenting any missing outcome data. We will note if both self-report and behavioural measures have been completed whilst wearing a hearing device. The authors will be contacted via email if sufficient detail is not reported. If data are only reported via figures, then WebPlotDigitizer (http:// arohatgi.info/ WebPlotDigitizer/ app/) will be used to extract data. A third reviewer will be involved if there is any disagreement between data extracted.

Study risk of bias assessment

Two reviewers will assess risk of bias for each study identified by for each study using the appropriate JBI critical appraisal tool. If disagreements arise a third reviewer will be involved. The JBI critical appraisal tools include assessment of methodological quality, and different checklists are used depending on the design of the study (e.g., cross sectional, longitudinal, randomized controlled trial). For each criteria, studies will be assessed for fulfilment (Yes, No, Unclear, or Not applicable).

Data synthesis

Key study characteristics will be described, including; study design, sample size, type of executive function measures used. The effect to be synthesised is the relationship between self-reported listening difficulties and behavioural measures of executive function defined by correlation coefficients. If correlation coefficients cannot be calculated or extracted for meta-analysis, study authors will be contacted to request the required information. Subgroup analyses will examine (where reported) key factors of, age, category/measure of self-reported listening difficulty, type of executive function measure, and type of hearing device, and pure-tone audiometric thresholds. Meta-analyses will be conducted for subgroups where data for a minimum of n=5 effects are available.

For peer review only - http://bmjopen.bmj.com/site/about/guidelines.xhtml

The meta-analysis will be carried out using the correlation coefficient as the outcome measure. A random-effects model will be fitted to the data. We will calculate at maximum one correlation coefficient per type of executive function by type of listening difficulty. If a study reports multiple different correlations, there is likely to be some level of dependency in the data that needs to be dealt with. To handle any dependency between effect sizes in the analyses, a multilevel random-effects meta-analysis approach, as recommended by Assink and Wibbelink, will be applied. [18] This approach includes one random effect for each study as an addition to the random effect for each effect size. Likelihood ratio tests will compare the fit of the multilevel model to the fit of the reduced models. If the multilevel random-effects analysis has better fit, it will be used in all analyses, otherwise the random effects model will be used.

The amount of heterogeneity (i.e., τ 2) will be estimated using the restricted maximum-likelihood estimator. [19] In addition to the estimate of τ 2, the Q-test for heterogeneity and the I^2 statistic will be reported. [20, 21] In case any amount of heterogeneity is detected (i.e., $\tau\hat{}2 > 0$, regardless of the results of the Q-test), a prediction interval for the true outcomes will also be provided [22]. Studentized residuals and Cook's distances will be used to examine whether studies can be defined as outliers and potentially influential in the context of the model. [23] Studies with a studentized residual larger than the $100 \times (1 - 0.05/(2 \times k))$th percentile of a standard normal distribution will be considered potential outliers (i.e., using a Bonferroni correction with two-sided $\alpha = 0.05$ for k studies included in the meta-analysis). Studies with a Cook's distance larger than the median plus six times the interquartile range of the Cook's distances will be considered influential. The analysis will be carried out using R and the metafor package. [24, 25]

The analysis for the secondary research question will be carried out in the same way as above, with the difference that meta-regressions will be used to investigate potential moderator effects. The moderators will be evaluated one by one, and if both get significant effects, they will be evaluated together. The meta regression procedure will follow the tutorial for meta regression on the metafor package home page. [26]

Reporting bias assessment

Funnel plots will be used to assess reporting bias. In addition, the funnel plot asymmetry will be evaluated with the rank correlation test and the regression test, using the standard error of the observed outcomes as predictor. [27, 28]

Ethics and Dissemination

This review does not raise any ethical issues. Results will be disseminated via scientific peer-reviewed journal articles, scientific magazines, and conference presentations.

Patient and Public Involvement

Patients or the public were not involved in the creation of this review protocol.

Study Design

MeSH terms will be used in relevant databases.

Table 2: Search terms for databases

**MEDLINE (OVID)** exp = explode the search term to include narrower more specific terms, .af. = search all fields in the document
1. exp Hearing Loss/

For peer review only - http://bmjopen.bmj.com/site/about/guidelines.xhtml

2.	exp Hearing/
3.	exp Self Report/
4.	(self report* or self-report* or questionnaire).af.
5.	exp Cognition/
6.	(cogniti* or executive or attention* or memory).af.
7.	(inhibit* or updat* or shift*) .af.
8.	1 or 2
9.	3 or 4
10.	5 or 6 or 7
11.	8 and 9 and 10

Author Contributions

HH, AH and HD developed the study. HH and AH created the search terms. JS, AH and HH wrote the review protocol and HD wrote the meta-analysis methods. AH, HD, EI, EH and LB provided critical feedback on drafts of the protocol.

Contact information

Jemaine E. Stacey
E-mail: Jemaine.stacey@ntu.ac.uk
Twitter: @DrJStacey

Henrik Danielsson
E-mail: henrik.danielsson@liu.se
Twitter: @henda52

Helen Henshaw
E-mail: helen.henshaw@nottingham.ac.uk
Twitter: @hlh1

Antje Heinrich
E-mail: antje.heinrich@manchester.ac.uk

Elisabeth Ingo
E-mail: elisabeth.ingo@liu.se

Emil Holmer
E-mail: emil.holmer@liu.se

Lucija Batinović
E-mail: lucija.batinovic@liu.se

Data statement

No datasets were generated or analysed for this protocol.

Funding

This study is funded by the National Institute for Health and Care Research (NIHR) [CDF-2018-11-ST2-016 and PB-PG-0816-20044], the NIHR Nottingham Biomedical Research Centre [BRC-1215-20003], the NIHR Manchester Biomedical Research Centre [NIHR BRC

For peer review only

For peer review only - http://bmjopen.bmj.com/site/about/guidelines.xhtml

20007] and by the Swedish Research Council grant 2017-06092. The views expressed are those of the author(s) and not necessarily those of the NIHR or the Department of Health and Social Care.

Competing interests

None declared

Acknowledgements: none.

Patient and Public involvement

Patients or the public were not involved in the creation of this protocol.

Word count: 2491 words

References

1. Dalton DS, Cruickshanks KJ, Klein BEK, *et al.* The Impact of Hearing Loss on Quality of Life in Older Adults. *Gerontologist*, **43(5):** 661–668. doi:10.1093/geront/43.5.661
2. Kiessling J, Pichora-Fuller MK, Gatehouse S, et al. Candidature for and delivery of audiological services: Special needs of older people. *Int J Audiol* 2003;**42(2)**:S92-S101. doi:10.3109/14992020309074650
3. Manchaiah VK, Stephens D. Perspectives on defining 'hearing loss' and its consequences. *Hearing Balance Commun* 2013;**11(1)**:6-16. doi:10.3109/21695717.2012.756624
4. Heinrich A, Henshaw H, Ferguson MA. Only behavioral but not self-report measures of speech perception correlate with cognitive abilities. *Front Psychol* 2016;**7**:576. doi:10.3389/fpsyg.2016.00576
5. Loughrey DG, Kelly ME, Kelley GA, *et al*. Association of age-related hearing loss with cognitive function, cognitive impairment, and dementia: A systematic review and meta-analysis. *JAMA Otolaryngol Head Neck Surg* 2018;**144(2)**:115-126. doi:10.1001/jamaoto.2017.2513
6. Marrone N, Ingram M, Bischoff K, *et al*. Self-reported hearing difficulty and its association with general, cognitive, and psychosocial health in the state of Arizona, 2015. *BMC Public Health* 2019;**19(1)**:875. doi:10.1186/s12889-019-7175-5
7. Livingston G, Huntley J, Sommerlad A, *et al*. Dementia prevention, intervention, and care: 2020 report of the Lancet Commission. *The Lancet* 2020;**396(10248)**:413-446. doi:10.1016/S0140-6736(20)30367-6
8. Heinrich A, Henshaw H, Ferguson MA. The relationship of speech intelligibility with hearing sensitivity, cognition, and perceived hearing difficulties varies for different speech perception tests. *Front Psychol* 2015;**6**:782. doi:10.3389/fpsyg.2015.00782
9. Friedman NP, Miyake A. Unity and diversity of executive functions: Individual differences as a window on cognitive structure. *Cortex* 2017;**86**:186-204. doi:10.1016/j.cortex.2016.04.023

For peer review only

For peer review only - http://bmjopen.bmj.com/site/about/guidelines.xhtml

10. Diamond A. Executive functions. *Annu Rev Psychol* 2013;**64**:135. doi:10.1146/annurev-psych-113011-143750
11. Miyake A, Friedman NP, Emerson MJ, *et al*. The unity and diversity of executive functions and their contributions to complex "frontal lobe" tasks: A latent variable analysis. *Cogn Psychol* 2000;**41(1)**:49-100. doi:10.1006/cogp.1999.0734
12. Fields TN, Mueller KD, Koscik RL, *et al*. Self-reported hearing loss and longitudinal cognitive function in a cohort enriched with risk for alzheimer's disease. *J Alzheimer's Dis* 2020;**78(3)**:1109-17. doi:10.3233/JAD-200701
13. Clarke NA, Henshaw H, Akeroyd MA, *et al*. Associations between subjective tinnitus and cognitive performance: systematic review and meta-analyses. *Trends Hear* 2020;**24**:2331216520918416. doi:10.1177/2331216520918416
14. Clarke NA, Akeroyd MA, Henshaw H, *et al*. The executive disruption model of tinnitus distress. *Front Psycho* in press.
15. Akeroyd MA, Wright-Whyte K, Holman JA, *et al*. A comprehensive survey of hearing questionnaires: how many are there, what do they measure, and how have they been validated?. *Trials* 2015;**16(1)**:1-1. doi:10.1186/1745-6215-16-S1-P26
16. Webb SL, Loh V, Lampit A, *et al*. Meta-analysis of the effects of computerized cognitive training on executive functions: a cross-disciplinary taxonomy for classifying outcome cognitive factors. *Neuropsychol Rev* 2018;**28(2)**:232-50. doi:10.1007/s11065-018-9374-8
17. World health organisation. Deafness and hearing loss. 2022. https://www.who.int/news-room/fact-sheets/detail/deafness-and-hearing-loss Accessed: 6 July 2022.
18. Assink M, Wibbelink CJ. Fitting three-level meta-analytic models in R: A step-by-step tutorial. *Quant Method Psychol* 2016;**12(3)**:154-74.
19. Viechtbauer W. Bias and efficiency of meta-analytic variance estimators in the random-effects model. *J Edu Behav Stat* 2005;**30(3)**:261-93. doi:10.3102/10769986030003261
20. Higgins JP, Thompson SG. Quantifying heterogeneity in a meta-analysis. *Stat Med* 2002;**21(11):**1539-58.
21. Cochran WG. (1954). The combination of estimates from different experiments. *Biometrics* 1954;**10(1)**:101–129. doi:10.2307/3001666
22. Riley RD, Higgins JP, Deeks JJ. Interpretation of random effects meta-analyses. *BMJ* 2011;**342**. doi:10.1136/bmj.d549
23. Viechtbauer W, Cheung MW. Outlier and influence diagnostics for meta-analysis. *Res Synth Methods* 2010;**1(2):**112-25. doi:10.1002/jrsm.11
24. R Core Team. R: A language and environment for statistical computing. R Foundation for Statistical Computing. 2021. www.R-project.org/
25. Viechtbauer, W. Conducting meta-analyses in R with the Metafor package. *J Stat Softw* 2010;**36(3)**:1-48.
26. Viechtbauer W. The Metafor Package: A meta-analysis package for R. Metafor Project. 2021. www.metafor-project.org/doku.php. Accessed: 6 July 2022.
27. Begg CB, Mazumdar M. Operating characteristics of a rank correlation test for publication bias. *Biom* 1994;**1**:1088-1101. doi:10.2307/2533446

For peer review only - http://bmjopen.bmj.com/site/about/guidelines.xhtml

28. Sterne JA, Egger M. Regression methods to detect publication and other bias in meta-analysis. In: Rothstein HR, Sutton AJ, Borenstein M, eds. *Publication bias in meta-analysis: Prevention, assessment and adjustments*. Wiley 2005. 99-110. doi:10.1002/0470870168

For peer review only

For peer review only - http://bmjopen.bmj.com/site/about/guidelines.xhtml

PRISMA-P (Preferred Reporting Items for Systematic review and Meta-Analysis Protocols) 2015 checklist: recommended items to address in a systematic review protocol*

Section and topic	Item No	Checklist item
**ADMINISTRATIVE INFORMATION**		
Title:		
Identification	1a	Identify the report as a protocol of a systematic review see title
Update	1b	If the protocol is for an update of a previous systematic review, identify as such n/a
Registration	2	If registered, provide the name of the registry (such as PROSPERO) and registration number see final line of abstract
Authors:		
Contact	3a	Provide name, institutional affiliation, e-mail address of all protocol authors; provide physical mailing address of corresponding author see both author affiliations and 'contact information' provided on page 8
Contributions	3b	Describe contributions of protocol authors and identify the guarantor of the review see 'author contributions' page 8 lines 4-7
Amendments	4	If the protocol represents an amendment of a previously completed or published protocol, identify as such and list changes; otherwise, state plan for documenting important protocol amendments N/A
Support:		
Sources	5a	Indicate sources of financial or other support for the review see 'funding' page 8 lines 37-43
Sponsor	5b	Provide name for the review funder and/or sponsor see 'funding' page 8 lines 37-43
Role of sponsor or funder	5c	Describe roles of funder(s), sponsor(s), and/or institution(s), if any, in developing the protocol page 8 lines 37-43
**INTRODUCTION**		
Rationale	6	Describe the rationale for the review in the context of what is already known page 2 line 21 onwards
Objectives	7	Provide an explicit statement of the question(s) the review will address with reference to participants, interventions, comparators, and outcomes (PICO) see page 4 'review questions' line 41 onwards AND the PICO is on page 5 lines 9-35
**METHODS**		
Eligibility criteria	8	Specify the study characteristics (such as PICO, study design, setting, time frame) and report characteristics (such as years considered, language, publication status) to be used as criteria for eligibility for the review see page 5 'eligibility criteria'
Information sources	9	Describe all intended information sources (such as electronic databases, contact with study authors, trial registers or other grey literature sources) with planned dates of coverage see 'information sources' page 5-6 line 43 onwards
Search strategy	10	Present draft of search strategy to be used for at least one electronic database, including planned limits, such that it could be repeated see table 1 on page 8-9
Study records:		

For peer review only

Data management	11a	Describe the mechanism(s) that will be used to manage records and data throughout the review page 6 lines 14-15
Selection process	11b	State the process that will be used for selecting studies (such as two independent reviewers) through each phase of the review (that is, screening, eligibility and inclusion in meta-analysis) see page 6 see "article selection process'
Data collection process	11c	Describe planned method of extracting data from reports (such as piloting forms, done independently, in duplicate), any processes for obtaining and confirming data from investigators see page 6 see "data extraction process"
Data items	12	List and define all variables for which data will be sought (such as PICO items, funding sources), any pre-planned data assumptions and simplifications page 6 see "data items"
Outcomes and prioritization	13	List and define all outcomes for which data will be sought, including prioritization of main and additional outcomes, with rationale page 5 23-27
Risk of bias in individual studies	14	Describe anticipated methods for assessing risk of bias of individual studies, including whether this will be done at the outcome or study level, or both; state how this information will be used in data synthesis see page 6 lines 32-35
Data synthesis	15a	Describe criteria under which study data will be quantitatively synthesised see data synthesis page 6 lines 38- page 7 line 1 onward
	15b	If data are appropriate for quantitative synthesis, describe planned summary measures, methods of handling data and methods of combining data from studies, including any planned exploration of consistency (such as I^2, Kendall's τ) page 7 lines 10-27
	15c	Describe any proposed additional analyses (such as sensitivity or subgroup analyses, meta-regression) page 6 lines 45-46
	15d	If quantitative synthesis is not appropriate, describe the type of summary planned page 2 line 1
Meta-bias(es)	16	Specify any planned assessment of meta-bias(es) (such as publication bias across studies, selective reporting within studies) see page 7 lines 30-32
Confidence in cumulative evidence	17	Describe how the strength of the body of evidence will be assessed (such as GRADE) see page 6 lines 32-35

*** It is strongly recommended that this checklist be read in conjunction with the PRISMA-P Explanation and Elaboration (cite when available) for important clarification on the items. Amendments to a review protocol should be tracked and dated.** **The copyright for PRISMA-P (including checklist) is held by the PRISMA-P Group and is distributed under a Creative Commons Attribution Licence 4.0.**

From: Shamseer L, Moher D, Clarke M, Ghersi D, Liberati A, Petticrew M, Shekelle P, Stewart L, PRISMA-P Group. Preferred reporting items for systematic review and meta-analysis protocols (PRISMA-P) 2015: elaboration and explanation. BMJ. 2015 Jan 2;349(jan02 1):g7647.

For peer review only

BMJ Open

The relationship between self-reported listening and communication difficulties and executive function: A protocol for a systematic review and meta-analysis

Journal:	*BMJ Open*
Manuscript ID	bmjopen-2022-071225.R2
Article Type:	Protocol
Date Submitted by the Author:	26-Sep-2023
Complete List of Authors:	Stacey, Jemaine; Nottingham Trent University, Psychology<br>Danielsson, Henrik; Linköping University, Department of Behavioural Sciences and Learning<br>Heinrich, Antje ; The University of Manchester<br>Batinović, Lucija; Linköping University, Department of Behavioural Sciences and Learning<br>Holmer, Emil ; Linköping University, Department of Behavioural Sciences and Learning<br>Ingo, Elisabeth; Linköping University, Department of Behavioural Sciences and Learning<br>Henshaw, Helen; University of Nottingham School of Medicine, Hearing Sciences, Mental Health and Clinical Neurosciences; NIHR Nottingham Biomedical Research Centre, National Institute for Health & Care Research (NIHR)
**Primary Subject Heading**:	Ear, nose and throat/otolaryngology
Secondary Subject Heading:	Geriatric medicine, Ear, nose and throat/otolaryngology, Communication
Keywords:	Audiology < OTOLARYNGOLOGY, GENERAL MEDICINE (see Internal Medicine), Aged

SCHOLARONE™
Manuscripts

The relationship between self-reported listening and communication difficulties and executive function: A protocol for a systematic review and meta-analysis

Jemaine E. Stacey[1] https://orcid.org/0000-0003-4035-712X Henrik Danielsson[2] https://orcid.org/0000-0002-0446-0827 Antje Heinrich[3] https://orcid.org/0000-0002-5940-622X Lucija Batinović[2], Emil Holmer[2] https://orcid.org/0000-0002-1896-8250, Elisabeth Ingo[2] https://orcid.org/0000-0003-4025-6345 and Helen Henshaw[4,5] https://orcid.org/0000-0002-0547-4403

[1]Nottingham Trent University, NTU Psychology, 50 Shakespeare St, Nottingham UK.
[2]Department of Behavioural Sciences and Learning, Linköping University, Linköping, Sweden.
[3]Manchester Centre for Audiology and Deafness (ManCAD), School of Health Sciences, University of Manchester, UK.
[4]National Institute for Health & Care Research (NIHR) Nottingham Biomedical Research Centre, Ropewalk House, 113 The Ropewalk, Nottingham, UK.
[5]Hearing Sciences, Mental Health and Clinical Neurosciences, School of Medicine, University of Nottingham, Nottingham, UK.

Keywords: hearing loss, listening and communication difficulties, self-report, cognition, executive function, memory, attention

Corresponding author: Dr Helen Henshaw, NIHR Nottingham Biomedical Research Centre, Ropewalk House, 113 The Ropewalk, Nottingham, NG1 5DU, UK.
Email: helen.henshaw@nottingham.ac.uk

Abstract

Introduction.
Listening and communication difficulties can limit people's participation in activity and adversely affect their quality of life. Hearing, as well as listening and communication difficulties can be measured either by using behavioural tests or self-report, and the outcomes are not always closely linked. The association between behaviourally measured and self-reported hearing is strong, whereas, the association between behavioural and self-reported measures of listening and communication difficulties is much weaker, suggesting they assess slightly different aspects of listening. While behavioural measures of listening and communication difficulties have been associated with poorer cognitive performance including executive functions, the same association has not always been shown for self-report measures. The objective of this systematic review and meta-analysis is to understand the relationship between executive function and self-reported listening and communication difficulties in adults with hearing loss, and where possible, potential covariates of age and pure-tone audiometric thresholds.

Methods and Analysis. Studies will be eligible for inclusion if they report data from both a self-report measure of listening difficulties and a behavioural measure of executive function. Eight databases are to be searched: MEDLINE (via Ovid SP), EMBASE (via Ovid SP), PsycINFO (via Ovid SP), ASSIA (via ProQuest), Cumulative Index to Nursing and Allied Health Literature or CINAHL (via EBSCO Host), Scopus, PubMed, and Web of Science (Science and Social Science Citation Index). The JBI critical appraisal tool will be used to assess risk of bias for included studies. Results will be synthesised primarily using a meta-

For peer review only

For peer review only - http://bmjopen.bmj.com/site/about/guidelines.xhtml

analysis, and where sufficient quantitative data are not available, a narrative synthesis will be carried out to describe key results. **Ethics and dissemination.** No ethical issues are foreseen. Data will be disseminated via academic publication and conference presentations. Findings may also be published in scientific newsletters and magazines. PROSPERO registration number CRD42022293546.

Article Summary

Strengths and limitations of this study

- This systematic review is the first to investigate the relationship between self-reported listening and communication difficulties and executive function using meta-analysis to synthesise the available evidence.
- It uses an established framework (ICF) and taxonomy (CHC-M) to define target domains and measures of listening and communication difficulties and executive functions respectively.
- Grey literature (including unpublished study results) will be included.
- This Protocol has been reported in accordance with the PRISMA-P statement
- Only studies available in English are eligible for inclusion

For peer review only

Introduction

Listening and communication are crucial for a healthy life and difficulties in communication can limit people's participation and adversely affect their quality of life. Hearing loss plays a fundamental role in determining a person's ability to listen and communicate, although research over the years has shown that there are other factors, beyond hearing loss, that are also important. Both hearing, and listening and communication, can be measured using behavioural measures such as pure-tone audiograms and speech-in-noise tests, or via self-report questionnaires. Behavioural and self-report measures of hearing are generally well correlated, and behavioural measures of hearing are often well correlated with both behavioural and self-report measures of listening and communication, i.e. questionnaires. When a listener has a behaviourally measured hearing loss, it is likely that they will also experience and report difficulties with listening and communicating.

Effective communication, which relies on good hearing, is instrumental for a high level of functioning and good quality of life. [1] Kiessling et al. proposed a cascade linking hearing to effective communication, which in turn can be mapped to the ICF Framework (core set for hearing loss). [2] Both frameworks are displayed in Table 1:

Table 1 Frameworks describing hearing and effective communication

**Kiessling et al. [2]**	**ICF Framework**	
	BODY FUNCTIONS<br>= physiological functions of body systems	
**Hearing:** a passive function that provides access to the auditory world via the perception of sound	b230	Hearing functions:
	ACTIVITIES AND PARTICIPATION	

For peer review only - http://bmjopen.bmj.com/site/about/guidelines.xhtml

	= execution of a task or action by an individual and involvement in a life situation	
**Listening:** the process of hearing with intention and attention	d115	Listening:
**Comprehending:** the reception of information, meaning or intent	d310	Communicating with – receiving – spoken messages
**Communication:** the bi-directional transfer of information, meaning or intent between two or more people	d350	Conversation:

On the other hand, considering the activities and participation domain of listening and communication, behavioural and self-report measures are less robustly correlated. This is highlighted by the fact that two individuals who experience the same pure-tone average audiometric thresholds can experience and report substantially different degrees of listening and communication difficulties [3]. One interpretation of this result could be that they assess slightly different concepts and or highlight different contributing factors. One of those contributing factors whose role still remains to be fully understood is cognition [4].

Cognition has a complex relationship with auditory function depending on whether it is considered on the function (hearing) or activities/participation (listening/communication) level. Specifically, hearing loss, both behaviourally measured and self-reported, has been shown to be associated with poorer cognitive performance across a range of cognitive domains including; global cognition, episodic memory, processing speed, semantic memory, visuospatial ability, executive functions, as well as cognitive impairment and dementia [5]. Indeed, Marrone and colleagues [6] reported that adults reporting any trouble hearing were at nearly four times higher odds of reporting increased confusion and memory loss and half as likely to report good general health compared to adults reporting no hearing difficulty. These results are important to acknowledge because hearing loss has been identified as the leading potentially modifiable risk factor for dementia in midlife [7].

For listening and communication, on the other hand, the type of assessment appears to play a role. For behavioural measures, the role of cognition for the ability to perceive speech (and in particular, speech in noise) has been reliably demonstrated for individuals with hearing loss, and this relationship is robust even when taking into consideration individuals' age and objective hearing levels (pure-tone average audiometric thresholds) [8]. Note that the cognitive ability most commonly assessed in studies of speech perception in noise is working memory. Other abilities such as attention and executive function are less regularly assessed and less robustly found to link to speech perception in noise. One reason for the less robust link might be that the speech in noise perception task needs to be a particular type or of more complexity in order to necessitate attentional and executive functions.

For self-report measures of listening or communication difficulties in quiet and in noise on the other hand the role of cognition is much less clear and a clear link with cognition is not always shown [8]. It is unclear why this link is so variable. Again, the cognitive ability most likely to be assessed is working memory. Maybe the listening situations most commonly assessed with self-report measures of communication are not of the type that require working memory or are more complex listening situations that would necessitate the involvement of executive functions. This idea would make sense given that listening and communicating in complex and noisy environments draws upon the ability to shut out distractions and maintain

For peer review only - http://bmjopen.bmj.com/site/about/guidelines.xhtml

focus. And thus it is conceivable that differences in *executive functions,* may play a key role in the variation of individual experiences of listening and communication difficulties, regardless of absolute hearing levels.

Executive functions refer to "higher order cognitive processes that control lower level cognitive processes in the service of goal-directed behaviour" (p.186). [9] They enable the ability to think before acting, plan, meet novel and unanticipated challenges, resist temptations and maintain focus. [10] According to Miyake and Friedman [11], there are three core executive functions: mental-set shifting (*shifting*), information updating and monitoring (*updating*), and inhibition of prepotent responses (*inhibition*). Indeed, there is emerging evidence from largescale Cohort studies that individuals with self-reported hearing loss exhibit significantly poorer performance on tests of flexibility, psychomotor speed and executive function. [12] Similarly, a systematic review of tinnitus research found individuals who reported tinnitus had poorer performance on measure of executive function compared with individuals who did not. [13] Subsequent empirical research showed that for a population of adults with tinnitus, those reporting that their tinnitus was bothersome showed poorer performance on measures of executive function compared with those reporting non-bothersome tinnitus. [14]

In this review, we aim to synthesise the evidence assessing the relationship between self-reported listening and communication difficulties and objective measures of executive function, whilst controlling (where possible) for the potentially confounding factors of age and pure-tone audiometric hearing thresholds. To our knowledge, this independent relationship has yet to be extensively examined, despite data pertaining to both executive functions and self-reported listening and communication difficulties often being reported as part of wider research study methods.

When considering measures of self-reported listening and communication difficulties, it is important to clearly define what we mean, as there are well over a hundred self-report measures pertaining to listening [15], and only a subset will be relevant to our current research question. For this reason, we adopt definitions of listening difficulties provided by the International Classification of Functioning and Disease (ICF) as *activity limitations* and *participation restrictions* arising from hearing loss, and narrow our focus to self-report measures that align with ICF core set for hearing loss domains of *listening* (d115), *communicating with – receiving – spoken messages* (d310) and *conversation* (d350). Similarly, to definitively identify executive function domains and classify behavioural measures of executive function as either shifting, updating or inhibition, we will use the Cattell-Horn-Carroll-Miyake (CHC-M) taxonomy. [16]

Review questions

Primary research question
Is there an association between self-reported listening and communication difficulties and performance on behavioural measures of executive function in adults with hearing loss?

Secondary research question
Is any association moderated by age and/or hearing loss (as measured using average pure-tone audiometric thresholds)?

For peer review only - http://bmjopen.bmj.com/site/about/guidelines.xhtml

Objectives

1. To review and synthesise evidence for the association between self-reported listening difficulties and performance on behavioural measures of executive function, in adults with hearing loss.

Methods and Analysis

Eligibility criteria

Participants

Adults with hearing loss (with or without hearing devices), aged 18 years and over with no reported cognitive decline. We will accept a qualitative definition of hearing loss as "mild', 'moderate', 'severe' or 'profound', or a quantitative definition where the group average pure-tone audiometric threshold is classed as mild hearing loss or greater using the World Health Organisation (WHO) definition of mild (26-40 dB HL inclusive); moderate (41-60 dB HL inclusive); severe (61-80 dB HL inclusive), and profound (81+ dB HL). [17] Studies that report on mixed populations (e.g. children & adults or normal hearing participants and participants with hearing loss) will be included only if the data for the populations of interest are reported separately.

Intervention/interest

A correlation coefficient between self-reported listening or communication difficulties and executive function, either reported or calculated from other reported data.

Outcomes

Self-reported listening and communication difficulties can be measured by a single item or a questionnaire assessing the following International Classification of Functioning and Disease (ICF) core set for hearing loss domains of listening (d115), communicating with – receiving – spoken messages (d310) and conversation (d350).

At least one behavioural measure of executive function must be included, defined according to the CHC-M taxonomy as tasks that measure: updating (e.g. verbal N-back), shifting (e.g. Trail making part B), and inhibition (e.g. Stroop). [16]

Where available, demographic information about the population (age, hearing device, group description) and objectively measured hearing loss (average pure-tone audiometric thresholds) will also be examined as subgroup descriptors and/or potential moderator(s).

Study design

Cross-sectional, longitudinal, experimental, quasi-experimental, and observational studies will be included.

Information sources

Articles must be available in English. No restrictions on publication dates will be applied.

Databases to be searched (see Table 2 for search terms): MEDLINE (via Ovid SP), EMBASE (via Ovid SP), PsycINFO (via Ovid SP), ASSIA (via ProQuest), Cumulative Index to Nursing and Allied Health Literature or CINAHL (via EBSCO Host), Scopus, PubMed, and Web of Science (Science and Social Science Citation Index). Gray literature including PhD theses, unpublished datasets and conference proceedings are eligible for inclusion.

For peer review only - http://bmjopen.bmj.com/site/about/guidelines.xhtml

Unpublished data will be accessed by contacting the corresponding authors of identified records. Literature searches were carried out on 11.05.2022.

Article selection process

Two reviewers will independently screen titles and abstracts, and full texts of retrieved records, against the inclusion and exclusion criteria. If insufficient information is provided in the titles and abstracts to know if it should be included or if there is disagreement between the two reviewers, the article will be included in the full-text screening. Disagreement at the full-text screening will be resolved by a third reviewer.

Data extraction process

A data extraction form will be created and improved by pilot testing before data extraction starts. The data from each study will be extracted separately by two reviewers and then compared. A third reviewer will be involved if there is any disagreement. Article selection and data extraction will be carried out using Covidence review management software (https://www.covidence.org/).

Data items

The data to be extracted are: the aim, study design, setting, conflicts of interest, demographic information about the population (age, hearing device, group description), sample size, bibliographic information (publication year, authors, journal), correlation coefficients between: self-reported listening difficulties and executive function (and [if reported] between pure-tone audiometric thresholds and self-reported listening difficulties/executive function), type of executive function measure, type of self-reported listening difficulty measure, and (where relevant) procedure of pure-tone audiometric assessment, as well as documenting any missing outcome data. We will note if both self-report and behavioural measures have been completed whilst wearing a hearing device. The authors will be contacted via email if sufficient detail is not reported. If data are only reported via figures, then WebPlotDigitizer (http:// arohatgi.info/ WebPlotDigitizer/ app/) will be used to extract data. A third reviewer will be involved if there is any disagreement between data extracted.

Study risk of bias assessment

Two reviewers will assess risk of bias for each study identified by for each study using the appropriate JBI critical appraisal tool. If disagreements arise a third reviewer will be involved. The JBI critical appraisal tools include assessment of methodological quality, and different checklists are used depending on the design of the study (e.g., cross sectional, longitudinal, randomized controlled trial). For each criteria, studies will be assessed for fulfilment (Yes, No, Unclear, or Not applicable).

Data synthesis

Key study characteristics will be described, including; study design, sample size, type of executive function measures used. The effect to be synthesised is the relationship between self-reported listening difficulties and behavioural measures of executive function defined by correlation coefficients. If correlation coefficients cannot be calculated or extracted for meta-analysis, study authors will be contacted to request the required information. Subgroup analyses will examine (where reported) key factors of, age, category/measure of self-reported listening difficulty, type of executive function measure, and type of hearing device, and pure-tone audiometric thresholds. Meta-analyses will be conducted for subgroups where data for a minimum of n=5 effects are available.

For peer review only - http://bmjopen.bmj.com/site/about/guidelines.xhtml

The meta-analysis will be carried out using the correlation coefficient as the outcome measure. A random-effects model will be fitted to the data. We will calculate at maximum one correlation coefficient per type of executive function by type of listening difficulty. If a study reports multiple different correlations, there is likely to be some level of dependency in the data that needs to be dealt with. To handle any dependency between effect sizes in the analyses, a multilevel random-effects meta-analysis approach, as recommended by Assink and Wibbelink, will be applied. [18] This approach includes one random effect for each study as an addition to the random effect for each effect size. Likelihood ratio tests will compare the fit of the multilevel model to the fit of the reduced models. If the multilevel random-effects analysis has better fit, it will be used in all analyses, otherwise the random effects model will be used.

The amount of heterogeneity (i.e., τ 2) will be estimated using the restricted maximum-likelihood estimator. [19] In addition to the estimate of τ 2, the Q-test for heterogeneity and the I^2 statistic will be reported. [20, 21] In case any amount of heterogeneity is detected (i.e., $\tau\hat{}2 > 0$, regardless of the results of the Q-test), a prediction interval for the true outcomes will also be provided [22]. Studentized residuals and Cook's distances will be used to examine whether studies can be defined as outliers and potentially influential in the context of the model. [23] Studies with a studentized residual larger than the $100 \times (1 - 0.05/(2 \times k))$th percentile of a standard normal distribution will be considered potential outliers (i.e., using a Bonferroni correction with two-sided $\alpha = 0.05$ for k studies included in the meta-analysis). Studies with a Cook's distance larger than the median plus six times the interquartile range of the Cook's distances will be considered influential. The analysis will be carried out using R and the metafor package. [24, 25]

The analysis for the secondary research question will be carried out in the same way as above, with the difference that meta-regressions will be used to investigate potential moderator effects. The moderators will be evaluated one by one, and if both get significant effects, they will be evaluated together. The meta regression procedure will follow the tutorial for meta regression on the metafor package home page. [26]

Reporting bias assessment

Funnel plots will be used to assess reporting bias. In addition, the funnel plot asymmetry will be evaluated with the rank correlation test and the regression test, using the standard error of the observed outcomes as predictor. [27, 28]

Ethics and Dissemination

This review does not raise any ethical issues. Results will be disseminated via scientific peer-reviewed journal articles, scientific magazines, and conference presentations.

Patient and Public Involvement

Patients or the public were not involved in the creation of this review protocol.

Study Design

MeSH terms will be used in relevant databases.

Table 2: Search terms for databases

A full search strategy is provided in the supplementary file

**MEDLINE (OVID)** exp = explode the search term to include narrower more specific terms, .af. = search all fields in the document

For peer review only - http://bmjopen.bmj.com/site/about/guidelines.xhtml

1.	exp Hearing Loss/
2.	exp Hearing/
3.	exp Self Report/
4.	(self report* or self-report* or questionnaire).af.
5.	exp Cognition/
6.	(cogniti* or executive or attention* or memory).af.
7.	(inhibit* or updat* or shift*) .af.
8.	1 or 2
9.	3 or 4
10.	5 or 6 or 7
11.	8 and 9 and 10

For peer review only

Author Contributions

HH, AH and HD developed the study. HH and AH created the search terms. JS, AH and HH wrote the review protocol and HD wrote the meta-analysis methods. AH, HD, EI, EH and LB provided critical feedback on drafts of the protocol.

Contact information

Jemaine E. Stacey
E-mail: Jemaine.stacey@ntu.ac.uk
Twitter: @DrJStacey

Henrik Danielsson
E-mail: henrik.danielsson@liu.se
Twitter: @henda52

Helen Henshaw
E-mail: helen.henshaw@nottingham.ac.uk
Twitter: @hlh1

Antje Heinrich
E-mail: antje.heinrich@manchester.ac.uk

Elisabeth Ingo
E-mail: elisabeth.ingo@liu.se

Emil Holmer
E-mail: emil.holmer@liu.se

Lucija Batinović
E-mail: lucija.batinovic@liu.se

Data statement

No datasets were generated or analysed for this protocol.

Funding

This study is funded by the National Institute for Health and Care Research (NIHR) [CDF-2018-11-ST2-016 and PB-PG-0816-20044], the NIHR Nottingham Biomedical Research

For peer review only - http://bmjopen.bmj.com/site/about/guidelines.xhtml

Centre [BRC-1215-20003], the NIHR Manchester Biomedical Research Centre [NIHR BRC 20007] and by the Swedish Research Council grant 2017-06092. The views expressed are those of the author(s) and not necessarily those of the NIHR or the Department of Health and Social Care.

Competing interests

None declared

Acknowledgements: none.

Patient and Public involvement

Patients or the public were not involved in the creation of this protocol.

Word count: 2491 words

References

1. Dalton DS, Cruickshanks KJ, Klein BEK, *et al.* The Impact of Hearing Loss on Quality of Life in Older Adults. *Gerontologist*, **43(5):** 661–668. doi:10.1093/geront/43.5.661
2. Kiessling J, Pichora-Fuller MK, Gatehouse S, et al. Candidature for and delivery of audiological services: Special needs of older people. *Int J Audiol* 2003;**42(2**):S92-S101. doi:10.3109/14992020309074650
3. Manchaiah VK, Stephens D. Perspectives on defining 'hearing loss' and its consequences. *Hearing Balance Commun* 2013;**11(1)**:6-16. doi:10.3109/21695717.2012.756624
4. Heinrich A, Henshaw H, Ferguson MA. Only behavioral but not self-report measures of speech perception correlate with cognitive abilities. *Front Psychol* 2016;**7**:576. doi:10.3389/fpsyg.2016.00576
5. Loughrey DG, Kelly ME, Kelley GA, *et al*. Association of age-related hearing loss with cognitive function, cognitive impairment, and dementia: A systematic review and meta-analysis. *JAMA Otolaryngol Head Neck Surg* 2018;**144(2)**:115-126. doi:10.1001/jamaoto.2017.2513
6. Marrone N, Ingram M, Bischoff K, *et al*. Self-reported hearing difficulty and its association with general, cognitive, and psychosocial health in the state of Arizona, 2015. *BMC Public Health* 2019;**19(1)**:875. doi:10.1186/s12889-019-7175-5
7. Livingston G, Huntley J, Sommerlad A, *et al*. Dementia prevention, intervention, and care: 2020 report of the Lancet Commission. *The Lancet* 2020;**396(10248)**:413-446. doi:10.1016/S0140-6736(20)30367-6
8. Heinrich A, Henshaw H, Ferguson MA. The relationship of speech intelligibility with hearing sensitivity, cognition, and perceived hearing difficulties varies for different speech perception tests. *Front Psychol* 2015;**6**:782. doi:10.3389/fpsyg.2015.00782
9. Friedman NP, Miyake A. Unity and diversity of executive functions: Individual differences as a window on cognitive structure. *Cortex* 2017;**86**:186-204. doi:10.1016/j.cortex.2016.04.023

For peer review only - http://bmjopen.bmj.com/site/about/guidelines.xhtml

10. Diamond A. Executive functions. *Annu Rev Psychol* 2013;**64**:135. doi:10.1146/annurev-psych-113011-143750
11. Miyake A, Friedman NP, Emerson MJ, *et al*. The unity and diversity of executive functions and their contributions to complex "frontal lobe" tasks: A latent variable analysis. *Cogn Psychol* 2000;**41(1)**:49-100. doi:10.1006/cogp.1999.0734
12. Fields TN, Mueller KD, Koscik RL, *et al*. Self-reported hearing loss and longitudinal cognitive function in a cohort enriched with risk for alzheimer's disease. *J Alzheimer's Dis* 2020;**78(3)**:1109-17. doi:10.3233/JAD-200701
13. Clarke NA, Henshaw H, Akeroyd MA, *et al*. Associations between subjective tinnitus and cognitive performance: systematic review and meta-analyses. *Trends Hear* 2020;**24**:2331216520918416. doi:10.1177/2331216520918416
14. Clarke NA, Akeroyd MA, Henshaw H, *et al*. The executive disruption model of tinnitus distress. *Front Psycho* in press.
15. Akeroyd MA, Wright-Whyte K, Holman JA, *et al*. A comprehensive survey of hearing questionnaires: how many are there, what do they measure, and how have they been validated?. *Trials* 2015;**16(1)**:1-1. doi:10.1186/1745-6215-16-S1-P26
16. Webb SL, Loh V, Lampit A, *et al*. Meta-analysis of the effects of computerized cognitive training on executive functions: a cross-disciplinary taxonomy for classifying outcome cognitive factors. *Neuropsychol Rev* 2018;**28(2)**:232-50. doi:10.1007/s11065-018-9374-8
17. World health organisation. Deafness and hearing loss. 2022. https://www.who.int/news-room/fact-sheets/detail/deafness-and-hearing-loss Accessed: 6 July 2022.
18. Assink M, Wibbelink CJ. Fitting three-level meta-analytic models in R: A step-by-step tutorial. *Quant Method Psychol* 2016;**12(3)**:154-74.
19. Viechtbauer W. Bias and efficiency of meta-analytic variance estimators in the random-effects model. *J Edu Behav Stat* 2005;**30(3)**:261-93. doi:10.3102/10769986030003261
20. Higgins JP, Thompson SG. Quantifying heterogeneity in a meta-analysis. *Stat Med* 2002;**21(11):**1539-58.
21. Cochran WG. (1954). The combination of estimates from different experiments. *Biometrics* 1954;**10(1)**:101–129. doi:10.2307/3001666
22. Riley RD, Higgins JP, Deeks JJ. Interpretation of random effects meta-analyses. *BMJ* 2011;**342**. doi:10.1136/bmj.d549
23. Viechtbauer W, Cheung MW. Outlier and influence diagnostics for meta-analysis. *Res Synth Methods* 2010;**1(2):**112-25. doi:10.1002/jrsm.11
24. R Core Team. R: A language and environment for statistical computing. R Foundation for Statistical Computing. 2021. www.R-project.org/
25. Viechtbauer, W. Conducting meta-analyses in R with the Metafor package. *J Stat Softw* 2010;**36(3)**:1-48.
26. Viechtbauer W. The Metafor Package: A meta-analysis package for R. Metafor Project. 2021. www.metafor-project.org/doku.php. Accessed: 6 July 2022.
27. Begg CB, Mazumdar M. Operating characteristics of a rank correlation test for publication bias. *Biom* 1994;**1**:1088-1101. doi:10.2307/2533446

For peer review only - http://bmjopen.bmj.com/site/about/guidelines.xhtml

28. Sterne JA, Egger M. Regression methods to detect publication and other bias in meta-analysis. In: Rothstein HR, Sutton AJ, Borenstein M, eds. *Publication bias in meta-analysis: Prevention, assessment and adjustments*. Wiley 2005. 99-110. doi:10.1002/0470870168

For peer review only

Search strategies
**MEDLINE & PsychINFO (OVID)** exp = explode the search term to include narrower more specific terms, .af. = search all fields in the document
1. exp Hearing Loss/<br>2. exp Hearing/<br>3. exp Self Report/<br>4. (self report* or self-report* or questionnaire).af.<br>5. exp Cognition/<br>6. cogniti* or executive or attention* or memory).af.<br>7. (inhibit* or updat* or shift*) .af.<br>8. 1 or 2<br>9. 3 or 4<br>10. 5 or 6 or 7<br>11. 8 and 9 and 10
**EMBASE (OVID)**
1. exp Hearing Disorders/<br>2. hearing.mp.<br>3. exp Self-Report/<br>4. exp Cognition/<br>5. 1 or 2<br>6. 3 and 4 and 5
**PubMed & Scopus**
1. hearing loss[MeSH Major Topic]<br>2. hearing[MeSH Major Topic]<br>3. self report[MeSH Major Topic]<br>4. self report*[Title/Abstract]<br>5. questionnaire[Title/Abstract]<br>6. cognition[MeSH Major Topic]<br>7. cogniti*[Title/Abstract]<br>8. executive[Title/Abstract]<br>9. attention*[Title/Abstract]<br>10. memory[Title/Abstract]<br>11. inhibi*[Title/Abstract]<br>12. updat*[Title/Abstract]<br>13. switch*[Title/Abstract]<br>14. 1 or 2<br>15. 3 or 4 or 5<br>16. 6 or 7 or 8 or 9 or 10 or 11 or 12 or 13<br>17. 14 and 15 and 16
**ASSIA (via ProQuest)**
1. su(Hearing Loss)<br>2. su(Hearing)<br>3. su(self-Report)<br>4. noft(self-report*)<br>5. noft(questionnaire)<br>6. su(cognition)<br>7. noft(cogniti*)<br>8. noft(executive)<br>9. noft(attention*)<br>10. noft(memory)<br>11. noft(inhibit*)

For peer review only

12. noft(update*)<br>13. noft(shift*)<br>14. 1 or 2<br>15. 3 or 4 or 5<br>16. 6 or 7 or 8 or 9 or 10 or 11 or 12 or 13<br>14. 14 and 15 and 16
**Web of Science**
1. (Hearing loss)<br>2. (Hearing)<br>3. (Self report)<br>4. KP=(Self report*)<br>5. KP=(questionnaire)<br>6. KP=(cogniti*))<br>7. KP=(attention*))<br>8. KP=(memory)<br>9. TS=(cognition)<br>10. 1 or 2<br>11. 3 or 4 or 5<br>12. 6 or 7 or 8 or 9<br>13. 10 and 11 and 12
**CINAHL (via EBSCO)**
1. MH hearing loss<br>2. deafness<br>3. hearing impairment<br>4. deaf<br>5. hard of hearing<br>6. MH self-report measures<br>7. self-report questionnaire<br>8. MH cognition<br>9. cognitive function<br>10. TX cogniti*<br>11. TX executive<br>12. TX attention*<br>13. TX memory<br>14. 1 or 2 or 3 or 4 or 5<br>15. 6 or 7<br>16. 8 or 9 or 10 or 11 or 12 or 13<br>17. 14 and 15 and 16

For peer review only

PRISMA-P (Preferred Reporting Items for Systematic review and Meta-Analysis Protocols) 2015 checklist: recommended items to address in a systematic review protocol*

Section and topic	Item No	Checklist item
**ADMINISTRATIVE INFORMATION**		
Title:		
Identification	1a	Identify the report as a protocol of a systematic review see title
Update	1b	If the protocol is for an update of a previous systematic review, identify as such n/a
Registration	2	If registered, provide the name of the registry (such as PROSPERO) and registration number see final line of abstract
Authors:		
Contact	3a	Provide name, institutional affiliation, e-mail address of all protocol authors; provide physical mailing address of corresponding author see both author affiliations and 'contact information' provided on page 8
Contributions	3b	Describe contributions of protocol authors and identify the guarantor of the review see 'author contributions' page 8 lines 4-7
Amendments	4	If the protocol represents an amendment of a previously completed or published protocol, identify as such and list changes; otherwise, state plan for documenting important protocol amendments N/A
Support:		
Sources	5a	Indicate sources of financial or other support for the review see 'funding' page 8 lines 37-43
Sponsor	5b	Provide name for the review funder and/or sponsor see 'funding' page 8 lines 37-43
Role of sponsor or funder	5c	Describe roles of funder(s), sponsor(s), and/or institution(s), if any, in developing the protocol page 8 lines 37-43
**INTRODUCTION**		
Rationale	6	Describe the rationale for the review in the context of what is already known page 2 line 21 onwards
Objectives	7	Provide an explicit statement of the question(s) the review will address with reference to participants, interventions, comparators, and outcomes (PICO) see page 4 'review questions' line 41 onwards AND the PICO is on page 5 lines 9-35
**METHODS**		
Eligibility criteria	8	Specify the study characteristics (such as PICO, study design, setting, time frame) and report characteristics (such as years considered, language, publication status) to be used as criteria for eligibility for the review see page 5 'eligibility criteria'
Information sources	9	Describe all intended information sources (such as electronic databases, contact with study authors, trial registers or other grey literature sources) with planned dates of coverage see 'information sources' page 5-6 line 43 onwards
Search strategy	10	Present draft of search strategy to be used for at least one electronic database, including planned limits, such that it could be repeated see table 1 on page 8-9
Study records:		

For peer review only

Data management	11a	Describe the mechanism(s) that will be used to manage records and data throughout the review page 6 lines 14-15
Selection process	11b	State the process that will be used for selecting studies (such as two independent reviewers) through each phase of the review (that is, screening, eligibility and inclusion in meta-analysis) see page 6 see "article selection process'
Data collection process	11c	Describe planned method of extracting data from reports (such as piloting forms, done independently, in duplicate), any processes for obtaining and confirming data from investigators see page 6 see "data extraction process"
Data items	12	List and define all variables for which data will be sought (such as PICO items, funding sources), any pre-planned data assumptions and simplifications page 6 see "data items"
Outcomes and prioritization	13	List and define all outcomes for which data will be sought, including prioritization of main and additional outcomes, with rationale page 5 23-27
Risk of bias in individual studies	14	Describe anticipated methods for assessing risk of bias of individual studies, including whether this will be done at the outcome or study level, or both; state how this information will be used in data synthesis see page 6 lines 32-35
Data synthesis	15a	Describe criteria under which study data will be quantitatively synthesised see data synthesis page 6 lines 38- page 7 line 1 onward
	15b	If data are appropriate for quantitative synthesis, describe planned summary measures, methods of handling data and methods of combining data from studies, including any planned exploration of consistency (such as I^2, Kendall's τ) page 7 lines 10-27
	15c	Describe any proposed additional analyses (such as sensitivity or subgroup analyses, meta-regression) page 6 lines 45-46
	15d	If quantitative synthesis is not appropriate, describe the type of summary planned page 2 line 1
Meta-bias(es)	16	Specify any planned assessment of meta-bias(es) (such as publication bias across studies, selective reporting within studies) see page 7 lines 30-32
Confidence in cumulative evidence	17	Describe how the strength of the body of evidence will be assessed (such as GRADE) see page 6 lines 32-35

*** It is strongly recommended that this checklist be read in conjunction with the PRISMA-P Explanation and Elaboration (cite when available) for important clarification on the items. Amendments to a review protocol should be tracked and dated.** **The copyright for PRISMA-P (including checklist) is held by the PRISMA-P Group and is distributed under a Creative Commons Attribution Licence 4.0.**

From: Shamseer L, Moher D, Clarke M, Ghersi D, Liberati A, Petticrew M, Shekelle P, Stewart L, PRISMA-P Group. Preferred reporting items for systematic review and meta-analysis protocols (PRISMA-P) 2015: elaboration and explanation. BMJ. 2015 Jan 2;349(jan02 1):g7647.

For peer review only
